# Experimental Testing of the Action of Vitamin D and Silicon Chelates in Bone Fracture Healing and Bone Turnover in Mice and Rats

**DOI:** 10.3390/nu14101992

**Published:** 2022-05-10

**Authors:** Aleksey Bychkov, Vyacheslav Koptev, Varvara Zaharova, Polina Reshetnikova, Elena Trofimova, Elena Bychkova, Ekaterina Podgorbunskikh, Oleg Lomovsky

**Affiliations:** 1 Scientific Department, Moscow State University of Food Production, 11 Volokolamskoe Hwy., 125080 Moscow, Russia; 2 Laboratory of Mechanochemistry, Institute of Solid State Chemistry and Mechanochemistry SB RAS, 18 Kutateladze, 630090 Novosibirsk, Russia; shapolova@solid.nsc.ru (E.T.); podgorbunskikh@solid.nsc.ru (E.P.); lomov@solid.nsc.ru (O.L.); 3 Laboratory of Young Animal Diseases, Siberian Federal Scientific Center of Agro-Biotechnologies RAS, 2b Centralnaya, 630501 Novosibirsk, Russia; kastrolog@mail.ru (V.K.); zaxarovabari@gmail.com (V.Z.); 4 Department of Business, Novosibirsk State Technical University, 20 Prospekt K. Marksa, 630073 Novosibirsk, Russia; reshetnikovapolina@ngs.ru (P.R.); bychkova.nstu@gmail.com (E.B.)

**Keywords:** silicon, silicon chelate, vitamin D, mechanochemistry, bone, fracture healing, bone loss, osteoporosis

## Abstract

This study presents findings on the biological action of an integrated supplement containing the following components involved in osteogenesis and mineralization: vitamin D and silicon in the bioavailable and soluble form. A hypothesis that these components potentiate one another’s action and make calcium absorption by the body more efficient was tested. Biological tests of the effect of vitamin D and silicon chelates on bone fracture healing and bone turnover were conducted using ICR mice and albino Wistar rats. Radiographic and biochemical studies show that the supplement simultaneously containing silicon chelates and vitamin D stimulates bone tissue regeneration upon mechanical defects and accelerates differentiation of osteogenic cells, regeneration of spongy and compact bones, and restoration of bone structure due to activation of osteoblast performance. Bone structure restoration was accompanied by less damage to skeletal bones, apparently due to better absorption of calcium from food. The studied supplement has a similar effect when used to manage physiologically induced decalcification, thus holding potential for the treatment of osteomalacia during pregnancy or occupational diseases (e.g., for managing bone decalcification in astronauts).

## 1. Introduction

An adequate vitamin D intake largely depends on person’s dietary intake, exposure to UV radiation, age, comorbidities, and concomitant use of some medications. Chronic and systemic vitamin D deficiency can increase the incidence of pathologies of many organs and organ systems in the human body [1,2], such as hypocalcemia, hyperparathyroidism, and hypophosphatemia (resulting in poor bone mineralization). Clinical manifestations of vitamin D deficiency also include age-independent fatigue and myalgia [3,4]. However, the functions of vitamin D are not confined exclusively to regulation of calcium and phosphorus turnover. This vitamin also affects other physiological processes occurring in the human body, such as cell growth modulation, neuromuscular transmission, and inflammatory processes. The most commonly reported sequelae of vitamin D deficiency include dysbasia, mobility and balance control impairment, as well as a higher incidence of falls, including those followed by fractures [5,6].

Unlike vitamin D, whose role in the human body has been investigated rather well, only a few studies point out the importance of compounds of silicon as an essential component for bone development. Some publications have showed that silicon is present in all body tissues, but the highest concentration of this element is found in bones, tendons, and other connective tissues. In vitro studies have demonstrated that silicon stimulates type 1 collagen synthesis and osteoblast differentiation [7]. Laboratory animal experiments have shown that silicon improves the incorporation of calcium into bones [8,9]. Silicon content in the osteoid is 25 times higher than that in the adjacent regions and gradually decreases with increasing calcification. One of the explanations of this fact is that silicon makes the bone surface more electronegative, and bone mineralization occurs in electronegative regions, emerging upon compression.

Animals receiving no silicon in their diets exhibit growth retardation and poor condition of connective tissue and bones [10]. Measurements of bone weight and strength in numerous model animals has showed the favorable effect of silicon supplements for increasing bone tissue density and reducing bone fragility [11].

Furthermore, silicon compounds can act as vehicles delivering the aforementioned biologically active substances [12,13]. This potential is related to the fact that silicon compounds are concentrated in tumors, wounds, and damaged muscle regions [14].

In most countries, there are no regulations for the dietary intake level of silicon and its content in foods; however, the boundary daily intake for children and adults was found to be 27 and 29 mg per day, respectively [15,16]. Insufficient intake of this element (daily intake < 5 mg) causes silicon deficiency.

Rice husks (or hulls) containing more than 20 wt. % of amorphous silicon dioxide are considered the most promising renewable source of biogenic silicon dioxide [17]. Silicon dioxide resides on the surface of rice husk particles and can be involved in complexation reactions with natural or artificially synthesized molecules carrying chelate groups [18]. Green tea biomass (leaves) containing eight different catechins carrying chelate groups is a promising feedstock from the perspective of the green chemistry concept [19].

Therefore, this study addresses the biological action of an integrated supplement simultaneously containing the following components involved in osteogenesis and mineralization: vitamin D and silicon in the bioavailable soluble form. It has been suggested earlier that these components are expected to potentiate one another’s action in a manner similar to the synergistic effects of vitamin D and vitamin K, which work together to activate osteocalcin [20]. The scarce and non-systematic findings that silicon compounds are needed to ensure proper and efficient calcium absorption by the human body attest to the validity of the tested hypothesis [21,22,23]. Mechanical activation was chosen as a technique for conducting the chelation reaction as it is an eco-friendly, waste-free and solution-free method for conducting a reaction between solid-phase inorganic, organic, and even polymeric reagents [24,25].

In this study, we have attempted to test the hypothesis that a supplement simultaneously containing silicon chelates and vitamin D reduces bone loss associated with damage and calcium deficiency. The results of this study involving laboratory animals could be useful for the control and treatment of physiologically induced decalcination (e.g., in pregnant women or astronauts).

## 2. Materials and Methods

### 2.1. Materials

The following materials and chemicals were used: rice husk *Oryza sativa (L.)* (Leader sort, Kyzylorda region, Kazakhstan), green tea *Camelia sinensis (L.)* (State standard No. TU 9191-003-00570186-04, Krasnodar, Russia), and 0.375 mg/mL aqueous solution of cholecalciferol (Grotesk Ltd., Moscow, Russia).

Moisture contents in the samples of plant raw materials and products were measured according to ref. [26] using a Radwag WPS 50SX automatic moisture analyzer (Radwag Uk Ltd., Radom, Poland) and were equal to 4.6%.

### 2.2. Chelated Silica

Chelated silica (silicon chelates) was produced mechanochemically using the previously described technology [27]. A mixture of 85% rice husk containing silica and 15% green tea containing catechins was subjected to mechanochemical activation in a RM-20 semi-industrial centrifugal roller type mill (manufactured at the Institute of Solid State Chemistry and Mechanochemistry, SB RAS, Novosibirsk, Russia) equipped with a water cooling system [28]. The technological regimes used in the work were as follows: feed rate, 50 kg/h; rotor speed, 1200 rpm. Temperature in the treatment zone was controlled by a water-cooled jacket and did not exceed 65–70 °C. Energy consumption of the centrifugal roller mill during mechanochemical activation of the reaction mixture was 10.3 kW. The resulting product was a grayish green fine powder, with the equilibrium concentration of silicon chelates in the solution equal to 20 mg/L.

### 2.3. Vitamin D Application

Vitamin D was coated onto the product containing silicon chelates at room temperature. For this purpose, 30 mL of 0.375 mg/mL cholecalciferol solution and then 5 mL of distilled water were slowly added dropwise to 30 g of the mechanochemically activated product under constant stirring. The resulting moist mixture was uniformly distributed over a freeze dryer tray and frozen at −18 °C. After freezing, the sample was dried in an Iney-4 freeze dryer (manufactured at the Institute for Biological Instrumentation, Pushchino, Moscow region, Russia). The condenser temperature was −47.3 °C; pressure above the sample at the end of the drying was 20 mtorr. The resulting product containing vitamin D and silicon chelates was ground in a mortar, stored in a vacuum pack at −18 °C, and used for further experiments.

### 2.4. Animals and Diets

Twelve-week-old pregnant female ICR mice (*n* = 45; weight range, 30–45 g) and twenty-four-week-old male albino Wistar rats (*n* = 45; weight range, 300–350 g) from the vivarium of the Siberian Federal Scientific Center of Agro-Biotechnologies RAS (Krasnoobsk, Russia) were used. The animals were euthanized in compliance with the requirements of the AVMA Guidelines for the Euthanasia of Animals (2013) by overdosing an inhalant anesthetic agent (anesthetic ether). All studies were conducted in compliance with the principles of humane treatment of laboratory animals in accordance with the requirements of the European Convention for the Protection of Vertebrate Animals used for Experimental and other Scientific Purposes, and Directive 2010/63/EU of the European Parliament and the Council of the European Union on the Protection of Animals used for Scientific Purposes. The animals were kept and cared for in accordance with GOST 33216-2014.

ICR mice received 15 g and Wistar rats received 60 g of compound feed manufactured by Delta Feeds (JSC BioPro, Novosibirsk, Russia) (Table 1). Animals in study group 1 received food supplemented with 2% of the product containing silicon chelates. Animals in study group 2 received food supplemented with 2% of the product containing silicon chelates and vitamin D. Animals in the control group received food without any supplements.

### 2.5. Formation of a Femoral Defect in Rats

Male Wistar rats were subjected to femoral drilling (formation of a defect in the femoral bone when modeling the mechanical damage to the bone tissue). Xyiazinum (Interchemie werken “De Adelaar” B.V., Venray, the Netherlands), at a dose of 0.05 mg/kg body weight, was used as an agent for induction of anesthesia. Zoletil 50 (Virbac S.A., Carro, France) at a dose of 0.5 mg/kg was used for the main course of anesthesia. After skin was dissected in the projection of *os femoris*, blunt dissection of the lateral condyle of *musculus quadratis femoris* was performed using Mosquito hemostatic forceps (Mopec Europe, Ltd., Oak Park, MI, USA) until the femoral shaft became visible in the wound. Next, the femoral wall was drilled in a direction perpendicular to the bone shaft using a sterile burr 1 mm in diameter. Wound debridement was performed and bone chips and blood clots were removed. The defect of the lateral condyle of *musculus quadratis femoris* was closed using a Π-shaped horizontal suture with 3-0 absorbable Vicryl Plus suture material (Ethicon Inc., Raritan, NJ, USA). The cutaneous wound was closed with interrupted sutures using Policon 2 suture material (tonzos 95 J.S.Co., Yambol, Bulgaria). The wound bed was managed twice daily with Terramycin (Zoetis Deutschland GmbH, Berlin, Germany).

### 2.6. Bone Fracture Healing 

Three groups (*n* = 15) of albino Wistar rats were formed using the analogy principle to study bone fracture healing. A 1 mm hole in the left femoral shaft was drilled in anesthetized animals in all the groups, with full compliance to the rules of aseptic and antiseptic surgery. The bone tissue had been exposed to mechanical damage before the analyzed supplements were added to the animals’ diet. On study day 21, the animals were euthanized, and blood and serum samples were collected to further perform morphological and biological studies. 

### 2.7. Physiological Bone Turnover 

Three groups (*n* = 15) of ICR mice in the last trimester of gestation were formed using the analogy principle to study the physiological bone turnover. On study day 21, the animals were euthanized, and blood and serum samples were collected to further perform morphological and biological studies. The animals’ thoracic spine, tail vertebrae, and femoral bones were also examined.

### 2.8. Blood Morphology and Serum Biochemistry Analyses

Blood morphology analysis was conducted using a Mindray BC-2800 Vet hematology analyzer (Mindray Medical International Ltd., Shenzhen, China). Serum biochemistry analysis was conducted on an URIT-800 Vet biochemistry analyzer (URIT Medical Electronic Co., Ltd., Shenzhen, China) using reagents produced by Joint-Stock Company Vital Development Corporation (St. Petersburg, Russia), Olvex Diagnosticum Ltd. (St. Petersburg, Russia), and Joint-Stock Company Vector-Best (Novosibirsk, Russia).

### 2.9. Bone X-ray

X-ray imaging of femoral bones was also performed using an AnyRay II X-ray machine (Vatech, Gyeonggi-do, Korea).

### 2.10. Statistics

The results are presented as the mean ± standard deviation (SD). The intergroup differences were tested for statistical significance using an unpaired Student’s *t*-test. A *p*-value < 0.05 was considered statistically significant, while *p*-values between 0.05 and 0.20 were noted as a trend.

## 3. Results

### 3.1. The Effect of Analyzed Supplements on Calcium Turnover in the Model of Mechanical Injury to Bone Tissue in Rats

**Figure 1:**
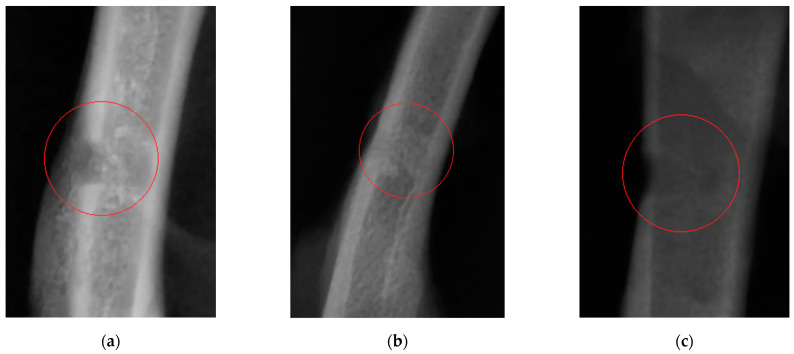
An X-ray image of the bone defect in rats on study day 21: (**a**) rats receiving the feed supplement containing silicon chelates; (**b**) rats receiving the feed supplement containing silicon chelates and vitamin D; and (**c**) rats receiving food without any supplements.

X-ray imaging on study day 21 (the time point at which femoral drilling was performed) clearly revealed the difference between bone tissue regeneration in the study and control groups (Figure 1).

The cortical index was calculated as follows [29]: 10 cm below the base of the lesser trochanter of the femoral bone, the total diameter of the bone diaphysis and the width of the medullary canal were measured. The difference between these two indicators corresponds to the total thickness of the cortical bone. The cortical thickness index is equal to the ratio between the cortical bone thickness and the total bone diameter (Table 2).

### 3.2. The Effect of Supplements on Morphological Parameters of Blood in Rats

Control blood sampling to analyze the blood morphology parameters was performed on day 21 after a hole in the rats’ bone was drilled and they started to receive feed supplements containing silicon chelates with and without vitamin D (Table 3). The blood level of PLT in rats in the control group was significantly increased compared to the normal range. The remaining morphology parameters lay within the normal range.

### 3.3. The Effect of Supplements Biochemical Parameters of Blood in Rats

The calcium/phosphorus ratio tended to decrease in group 2 compared to the control group. The activity of alkaline phosphatase was decreased in group 2 (Table 4).

### 3.4. The Effect of Supplements under Study in the Model of Physiological Decalcification of Bone Tissue in Mice

The experiment aiming to study the physiological decalcification was conducted in pregnant mice that were subdivided into three groups using the analogy principle. The morphological pattern of animals’ blood in the experiment is summarized in Table 5. The blood levels of PLT in mice in group 1 and the control group were significantly decreased compared to the normal range. The MCH level for all the groups was reduced compared to the normal value. The remaining morphology parameters lay within the normal range.

Table 6 summarizes the analyzed biochemical parameters of blood serum that support this assumption and agree with the findings revealed in the studies focusing on healing of mechanical damage to bones. Alkaline phosphatase activity for group 1 was significantly increased compared to the control group, while alkaline phosphatase activity tended to increase in group 2 compared to the control group.

### 3.5. The Effect of Supplements on Physiological Bone Turnover and Cortical Thickness Index in Mice

**Figure 2:**
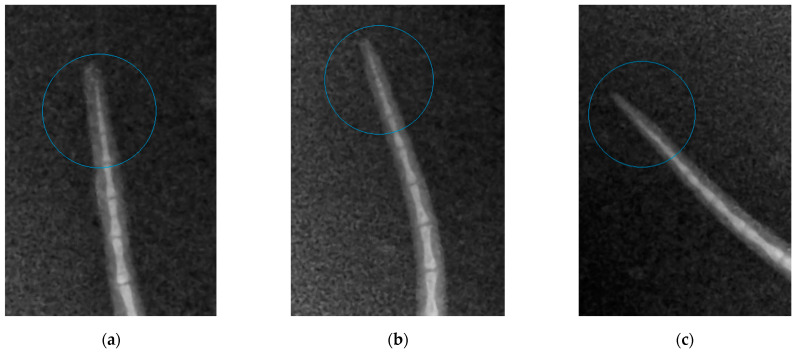
An X-ray image of tail vertebrae in mice: (**a**) mice receiving feed supplement containing silicon chelates (Group 1); (**b**) mice receiving feed supplement containing silicon chelates and vitamin D (Group 2); (**c**) mice receiving food without any supplements (Control group).

The radiographic findings are shown in Figure 2. An analysis of the X-ray images shows that the well-known decalcification and osteomalacia of the ultimate tail vertebra resulting from disturbance of calcium and phosphate metabolism are observed in the control group (in pregnant mice). The pattern of the vertebral body is vague because of low bone density; the boundaries between the bone and cartilaginous tissue are less defined compared to those in animals in the study groups.

**Figure 3:**
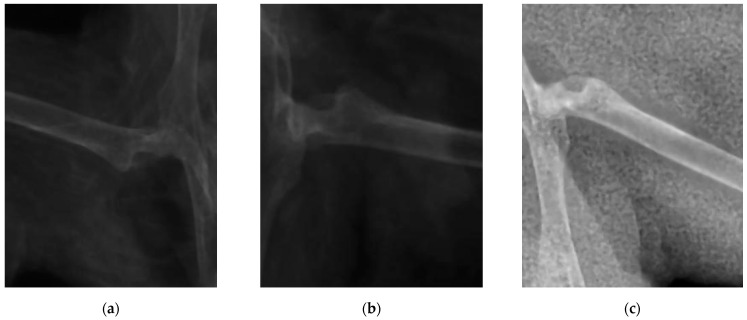
An X-ray image femoral bones in mice: (**a**) mice receiving feed supplement containing silicon chelates (Group 1); (**b**) mice receiving feed supplement containing silicon chelates and vitamin D (Group 2); (**c**) mice receiving food without any supplements (Control group).

A similar situation is revealed by analyzing the X-ray images of femoral bones (Figure 3) and calculating the cortical thickness index (Table 7).

## 4. Discussion

This study has addressed the potential therapeutic effect of an integrated feed supplement simultaneously containing silicon chelates and vitamin D on healing of bone fractures and bone regeneration in mice and rats.

Wistar rats are the main model organism for conducting experiments focusing on the effect of feed supplements on the animal and human bodies. ICR mice are often used for toxicology studies, as well as studies related to aging and the overall effect of supplements on the animal body. Pregnant mice were selected to optimally model physiological decalcification of tubular bones in animals and humans during pregnancy.

Changes in the morphological and biochemical composition of animals’ blood, visible signs of bone decalcification, the pattern of bone defect healing, the level of callus calcification, and the difference in cortical thickness indices between animals in the study groups and control group were used as assessment criteria.

Twenty-one days after the experiment start in Wistar rats (the time point when femoral drilling was performed and the analyzed feed supplements started to be received), the clinical condition of two rats in the control group worsened (as severely as bone fracturing in the defect area), while animals in the study groups did not have these complications. Partial ossification of osteoid tissue and the beginning of tubular bone structure restoration (in particular, cortical bone regeneration) were observed in study group 1. Complete regeneration of compact bone, full callus resorption, and activated bone structure restoration were revealed in study group 2 animals. A clear boundary of cortical bone restoration can be seen. The control group was characterized by incomplete restoration of the tubular bone structure and fragmentary replacement of the cartilaginous callus. The cortical bone was not restored; defect margins are well-marked. Consistent with the radiographic findings, morphological parameters of blood showed secondary erythrocytosis and thrombocytosis, which are typical of post-traumatic conditions in animals. In Wistar rats receiving the feed supplement simultaneously containing vitamin D and chelated silicon (group 2), unlike the same parameters in rats in study group 1 and the control group, the Ca/P ratio lay within the physiological range [33], which indicates that bone tissue regeneration was completed and mineral turnover in the body was normalized. The effectiveness of the diet containing the designed feed supplement has also been proved according to the reduced activity of alkaline phosphatase compared to that in the control group. The cortical thickness index in animals in the study groups on study day 21 was closer to the limits of the normal range, compared to this parameter in the control group. If the cortical thickness index is smaller than the normal value [29], it is fair to say that cortical bone thinning and calcium depletion has taken place. Hence, it has been demonstrated that the use of the supplements under study favorably affects calcium and phosphorus absorption from food and prevents bone decalcification during bone regeneration.

As follows from the reported data (the experiment involving pregnant ICR mice), the analyzed supplements containing silicon chelates and vitamin D exhibited no prominent effect on the morphological composition of blood. Most parameters either lay within the physiological normal range or beyond it with no statistically significant intergroup differences. However, it is noteworthy that in animals in study group 2, who received food supplemented with vitamin D and silicon chelates, the platelet count (PLT) lay within the physiological normal range, unlike in the study group 1 and control group animals who had severe thrombocytopenia. This condition can be either idiopathic or related to dysmetabolism caused by unbalanced nutrition. In all the groups, the cortical thickness index was decreased compared to the normal range, owing to physiological decalcification of tubular bones during pregnancy. Meanwhile, in animals in study group 2 that received the analyzed feed supplement simultaneously containing silicon chelates and vitamin D_3_, this parameter was 1.79% higher than the control value, which may indicate that the supplement had a weak stimulating action on calcium turnover. Our findings imply that the feed supplement containing silicon chelates and vitamin D improves calcium availability in foods during physiological bone tissue decalcification, which can be seen as a 11.9% increase in serum calcium level, while the rate of osteomalacia processes decreases simultaneously.

## 5. Conclusions

Study results suggest that the integrated supplement simultaneously containing silicon chelates and vitamin D stimulates bone regeneration after mechanical defects and accelerates differentiation of osteogenic cells and regeneration of spongy and compact bone, as well as restoration of bone structure due to stimulation of osteoblast performance. Bone structure restoration is accompanied by a smaller damaging impact on the skeletal system due to a reduced level of decalcification. The studied supplement has a similar effect when used to manage physiologically induced decalcification, thus holding potential for the treatment of osteomalacia during pregnancy or occupational diseases (e.g., for managing bone decalcification in astronauts).

## Figures and Tables

**Table 1:** The composition of diets used to study the effect of supplements on bone turnover in animals with physiologically induced decalcification and injuries. Animals in the control group received food without any supplements.

	Control Group		Group 1		Group 2	
	Wistar Rats	ICR Mice	Wistar Rats	ICR Mice	Wistar Rats	ICR Mice
Metabolizable energy (kcal/100 g)	322.4	310.5	322.4	310.5	322.4	310.5
Crude protein (wt.%)	22	19	22	19	22	19
Crude fiber (wt.%)	5	4	5	4	5	4
Crude fat (wt.%)	5	4	5	4	5	4
Crude ash (wt.%)	8	6	8	6	8	6
Silicon chelates (mg/g diet)	-	-	0.4	0.4	0.39	0.39
Vitamin D (mg/g diet)	-	-	-	-	0.0075	0.0075

Control group: Normal control diet (Delta Feeds, ICR mice received 15 g and Wistar rats received 60 g); Group 1: Control diet supplemented with silicon chelates, 2%; Group 2: Control diet supplemented with silicon chelates and vitamin D, 2%.

**Table 2:** The cortical thickness index of rats receiving the feed supplement containing chelated silicon and vitamin D after the experimentally induced mechanical damage to the femoral bone.

Group	Cortical Thickness Index (%)	Normal Range [29]
Rats receiving the feed supplement containing silicon chelates (Group 1)	45.60 ± 7.92	≥54%
Rats receiving the feed supplement containing silicon chelates and vitamin D (Group 2)	43.75 ± 2.69 *	
Rats receiving food without any supplements (Control group)	34.30 ± 6.85 *	

Data are presented as mean ± SD. * Significant differences between group 2 and control group compared to the normal range (*p*-value < 0.01).

**Table 3:** The blood morphology parameters of rats receiving feed supplements containing chelated silicon and vitamin D after the experimentally induced mechanical damage to the femoral bone.

Parameter	Rats Receiving the Feed Supplement Containing Silicon Chelates (Group 1)	Rats Receiving the Feed Supplement Containing Silicon Chelates and Vitamin D (Group 2)	Rats Receiving Food without any Supplements (Control Group)	Normal Range [30]
WBC × 10^9^/L	5.075 ± 1.9	6.975 ± 4.23	9.34 ± 3.1	2.9–15.3
Lymph × 10^9^/L	3.025 ± 0.46	3.775 ± 1.66	6.06 ± 3.82	2.6–13.5
Mon × 10^9^/L	0.225 ± 0.22	0.45 ± 0.44	0.42 ± 0.21	0.0–0.5
Gran × 10^9^/L	1.825 ± 1.35	2.75 ± 2.25	2.86 ± 2.3	0.4–3.2
Lymph (%)	63.175 ± 15.6	58.575 ± 10.49	62.7 ± 23.8	63.7–90.4
Mon (%)	4.575 ± 2.13	5.625 ± 2.13	4.8 ± 2.19	1.5–4.5
Gran (%)	32.25 ± 13.55	35.8 ± 8.43	44.56 ± 21.14	7.3–30.1
RBC × 10^12^/L	9.738 ± 0.75	9.3075 ± 0.38	8.784 ± 0.88	5.6–7.89
HGB (g/L)	139.2 ± 8.52	144.75 ± 9.94	130.4 ± 5.85	120–150
HCT (%)	44.92 ± 2.92	46.175 ± 3.17	41.66 ± 2.34	36.0–46.4
MCV (fL)	46.24 ± 2.02	49.725 ± 3.87	47.7 ± 2.6	53.0–68.8
MCH (pg)	14.3 ± 0.96	15.55 ± 1.04	14.84 ± 0.93	16.0–23.2
MCHC (g/L)	309.4 ± 4.72	314 ± 6.16	312.8 ± 5.44	300–341
RDW (%)	12.14 ± 0.81	12.35 ± 1.26	14.42 ± 1.45	11.0–15.2
PLT × 10^9^/L	425.4 ± 357.2	561.75 ± 304.01 *	722.2 ± 117.95 **	100–161
MPV (fL)	5.1 ± 0.64	5.05 ± 0.73	5.02 ± 0.57	3.8–6.2
PDW	16.14 ± 0.33	16.275 ± 0.74	16.08 ± 0.55	n.d.
PCT (%)	0.2044 ± 0.17	0.26875 ± 0.13	0.2846 ± 0.14	n.d.

Data are presented as mean ± SD. WBC, white blood cells; Lymph, lymphocytes; Mon, monocytes; Gran, granulocytes; RBC, red blood cells; HGB, hemoglobin, HCT, hematocrit; MCV, mean corpuscular volume; MCH, mean corpuscular hemoglobin; MCHC, mean corpuscular hemoglobin concentration; RDW, red cell distribution width; PLT, platelets; MPV, mean platelet volume; PDW, platelet distribution width; PCT, plateletcrit. * Tendency to increase compared to the normal range (*p*-value < 0.2). ** Significant differences compared to normal range (*p*-value < 0.001).

**Table 4:** Blood biochemistry parameters for serum samples of rats receiving feed supplements containing chelated silicon and vitamin D after the experimentally induced mechanical damage to the femoral bone.

Parameter	Rats Receiving the Feed Supplement Containing Silicon Chelates (Group 1)	Rats Receiving the Feed Supplement Containing Silicon Chelates and Vitamin D (Group 2)	Rats Receiving Food without Any Supplements (Control Group)
Calcium	2.03 ± 0.14	2.16 ± 0.28	2.01 ± 0.16
Phosphorus	1.52 ± 0.45	2.54 ± 1.23	1.39 ± 0.15
Calcium/Phosphorus	1.38 ± 0.35	0.99 ± 0.21 *	1.46 ± 0.21
Magnesium	0.76 ± 0.005	0.76 ± 0.05	0.78 ± 0.008
Alkaline phosphatase	674.1 ± 119.5	481.7 ± 147.5 *	914.02 ± 163.3

Data are presented as mean ± SD. * Tendency to decrease compared to the control group (*p*-value < 0.1).

**Table 5:** The blood morphology parameters of ICR mice receiving feed supplements based on chelated silicon and vitamin D upon physiological bone decalcification.

Parameter	Mice Receiving Feed Supplement Containing Silicon Chelates (Group 1)	Mice Receiving Feed Supplement Containing Silicon Chelates and Vitamin D (Group 2)	Mice Receiving Food without Any Supplements (Control Group)	Normal Range [31,32]
WBC × 10^9^/L	4.3 ± 1.45	5.3 ± 2.4	4.62 ± 2.03	0.8–6.8
Lymph × 10^9^/L	2.46 ± 0.96	3.28 ± 2.5	2.78 ± 1.09	0.7–5.7
Mon × 10^9^/L	0.2 ± 0.1	0.24 ± 0.16	0.24 ± 0.19	0.0–0.3
Gran × 10^9^/L	1.64 ± 0.48	1.78 ± 0.7	1.6 ± 0.9	0.1–1.8
Lymph (%)	56.76 ± 6.22	55.8 ± 19.5	60.84 ± 11.2	55.8–90.1
Mon (%)	4.76 ± 1.77	5.26 ± 2.81	4.98 ± 2.1	1.8–6.0
Gran (%)	38.48 ± 4.75	38.94 ± 17.3	34.18 ± 9.1	8.6–38.5
RBC × 10^12^/L	8.614 ± 0.8	8.506 ± 0.99	8.242 ± 0.56	6.36–9.4
HGB (g/L)	112.6 ± 14.8	95.34 ± 53.9	115.6 ± 9.96	110–143
HCT (%)	38.16 ± 5.27	38.96 ± 4.99	38.72 ± 3.16	34.6–44.8
MCV (fL)	44.34 ± 4.26	45.86 ± 1.74	47 ± 1.2	48.2–58.1
MCH (pg)	13.02 ± 0.97	13.62 ± 0.3	13.96 ± 0.2	15.8–19.4
MCHC (g/L)	294.8 ± 12.07	298.6 ± 10.71	298.2 ± 6.9	302–354
RDW (%)	20.44 ± 3.42	17.62 ± 1.68	18.38 ± 2.36	13.0–17.0
PLT × 10^9^/L	394.6 ± 29.67 *	482.4 ± 32.76	335.5 ± 13.24 **	450–1530
MPV (fL)	5.106 ± 0.24	4.98 ± 0.21	5.12 ± 0.36	3.8–6.0
PDW	15.646 ± 0.45	15.94 ± 0.41	15.98 ± 0.2	n.d.
PCT (%)	0.1988 ± 0.12	0.236 ± 0.16	0.239 ± 0.16	n.d.

Data are presented as mean ± SD. WBC, white blood cells; Lymph, lymphocytes; Mon, monocytes; Gran, granulocytes; RBC, red blood cells; HGB, hemoglobin, HCT, hematocrit; MCV, mean corpuscular volume; MCH, mean corpuscular hemoglobin; MCHC, mean corpuscular hemoglobin concentration; RDW, red cell distribution width; PLT, platelets; MPV, mean platelet volume; PDW, platelet distribution width; PCT, plateletcrit. * Tendency to decrease compared to the normal range (*p*-value < 0.1). ** Significant differences compared to normal range (*p*-value < 0.001).

**Table 6:** Biochemistry parameters of blood serum samples from ICR mice receiving feed supplements containing chelated silicon and vitamin D upon physiological bone decalcification.

Parameter	Mice Receiving Feed Supplement Containing Silicon Chelates	Mice Receiving Feed Supplement Containing Silicon Chelates and Vitamin D (Group 2)	Mice Receiving Food without Any Supplements (Control Group)
Calcium	2.26 ± 0.5	2.25 ± 0.21	2.01 ± 0.21
Phosphorus	2.27 ± 1.07	2.1 ± 0.22	2.29 ± 0.71
Calcium/Phosphorus	1.18 ± 0.54	1.08 ± 0.18	0.98 ± 0.44
Magnesium	0.76 ± 0.007	0.77 ± 0.05	0.77 ± 0.01
Alkaline phosphatase	409.78 ± 11.4 *	341.12 ± 91.38	174.8 ± 8.01

Data are presented as mean ± SD. * Significant differences compared to control group (*p*-value < 0.001).

**Table 7:** The cortical thickness index of ICR mice receiving feed supplement containing silicon chelates and vitamin D upon physiological decalcification of bone tissue.

Group	Cortical Thickness Index (%)	Normal Range [29]
Mice receiving feed supplement containing silicon chelates (Group 1)	26.12 ± 2.39 *	≥54%
Mice receiving feed supplement containing silicon chelates and vitamin D (Group 2)	31.25 ± 3.24 *	
Mice receiving food without any supplements (Control group)	30.70 ± 2.86 *	

Data are presented as mean ± SD. * Significant differences between the groups compared to the normal range *p*-value < 0.01.

## Data Availability

Not applicable.
